# Cerebral lesions sites in neurosarcoidosis: a lesion mapping study

**DOI:** 10.1007/s00415-023-11863-3

**Published:** 2023-07-12

**Authors:** Kilian Fröhlich, Anne Mrochen, Ruihao Wang, David Haupenthal, Kosmas Macha, Gabriela Siedler, Michael Knott, Arnd Dörfler, Stefan Schwab, Klemens Winder

**Affiliations:** 1 Department of Neurology, University Hospital Erlangen, Friedrich-Alexander University Erlangen-Nürnberg (FAU), Schwabachanlage 6, 91054 Erlangen, Germany; 2 Department of Neuroradiology, University Hospital Erlangen, Friedrich-Alexander University Erlangen-Nürnberg (FAU), Schwabachanlage 6, 91054 Erlangen, Germany

**Keywords:** Sarcoidosis, Neurosarcoidosis, Cerebral lesions, Voxel-based lesion symptom mapping

## Abstract

**Background and purpose:**

Sarcoidosis is a granulomatous disease of unknown etiology affecting the central nervous system in up to 15% of the patients. Diagnosis of neurosarcoidosis is very challenging due to the heterogeneity of its clinical manifestation. This study intended to evaluate the distribution of cerebral lesion sites and the potential presence of specific lesion clusters in neurosarcoidosis patients using voxel-based lesion symptom mapping (VLSM).

**Methods:**

Patients with neurosarcoidosis were retrospectively identified and included between 2011 and 2022. Cerebral lesion sites were correlated voxel-wise with presence and absence of neurosarcoidosis using non-parametric permutation test. Multiple sclerosis patients served as controls for the VLSM-analysis.

**Results:**

Thirty-four patients (mean age 52 ± 15 years) of whom 13 were diagnosed with possible, 19 with probable and 2 with confirmed neurosarcoidosis were identified. Lesion overlap of neurosarcoidosis patients demonstrated a distribution of white matter lesions in all brain areas, with a periventricular predilection similar to multiple sclerosis. In contrast to multiple sclerosis controls, no propensity for lesions in proximity of the corpus callosum was observed. Neurosarcoidosis lesions appeared smaller and lesion volume was lower in the neurosarcoidosis cohort. The VLSM analysis showed minor associations between neurosarcoidosis and damaged voxels in the bilateral frontobasal cortex.

**Conclusions:**

The VLSM analysis yielded significant associations in the bilateral frontal cortex, suggesting that leptomeningeal inflammatory disease with following cortical involvement is a quite specific feature in neurosarcoidosis. Lesion load was lower in neurosarcoidosis than in multiple sclerosis. However, no specific pattern of subcortical white matter lesions in neurosarcoidosis was revealed.

## Introduction

Sarcoidosis is a granulomatous multiorgan disease of unknown etiology affecting 1–40 in 10,000 persons [1]. Neurosarcoidosis has a prevalence of up to 15% in sarcoidosis patients [2], but autopsy studies suggest that numbers may be much higher as correct diagnosis is only made in 50% of the patients with nervous system involvement [3]. Neurological symptoms are very variable and may include every part of the nervous system [4, 5]. However, most commonly cranial neuropathies, meningitis, myelitis, hydrocephalus, and polyneuropathy are observed [2, 6].

Knowledge of neurosarcoidosis is highly relevant for various reasons. On the one hand, it has a severe impact on quality of life of patients because of the disablement due to the severity of symptoms, stressing the importance of an early diagnosis [1, 7, 8]. On the other hand, because of this heterogeneity of clinical manifestation, neurosarcoidosis is a very important differential diagnosis especially among inflammatory central nervous system (CNS) disorders [7, 9–11]. Diagnosis is challenging as there is no specific diagnostic parameter except histopathology via biopsy, which is only applied with reluctance in the CNS [2, 6, 12].

Although data on neurosarcoidosis is scarce, cerebral parenchymal lesions in neurosarcoidosis are predominantly located in the white matter and those lesions are regarded as the probably most frequent, but usually unspecific CNS affection [7, 13, 14]. However, subcortical vascular lesions are a frequent phenomenon in the elderly population and therefore discrimination between presumable inflammatory or vascular etiology is hardened [15]. Additionally, lesion patterns with predilection of periventricular and juxtacortical brain regions exist in some inflammatory CNS conditions like multiple sclerosis (MS) and need to distinguished from neurosarcoidosis parenchymal cerebral lesions, too [16].

To our knowledge, previous imaging studies in neurosarcoidosis only comprised descriptive case series [7, 10, 13, 14, 17–20]. Moreover, statistical imaging analysis like the voxel-wise lesion symptom mapping (VLSM) allows investigating voxel-by-voxel associations between cerebral lesion location and an outcome without having any a priori hypothesis.

Therefore, in the present study, we systematically and statistically assessed brain lesion sites in neurosarcoidosis patients. Using novel voxel-based lesion symptom mapping (VSLM) techniques to reveal possible specific lesion patterns in this condition, we tested the hypothesis whether lesion sites in the brain show a specific susceptibility for neurosarcoidosis.

## Patients and methods

### Patients

All medical records of patients admitted to the Department of Neurology at the University Hospital Erlangen are entered into a database. For the study, patients between 2011 and 01/2023 were screened. Patients were excluded if they had history of other cerebral conditions. The diagnostic workup followed the Consensus Criteria of the Neurosarcoidosis Consortium Consensus Group and therefore divided patients into definite, probable and possible neurosarcoidosis [5, 12]. Diagnosis of possible neurosarcoidosis included a clinical presentation suggestive for neurosarcoidosis, with exclusion of alternative diagnoses. For diagnosis of probable neurosarcoidosis, additional evidence of central nervous system inflammation in the cerebrospinal fluid, MRI or of systemic sarcoidosis was needed [5]. For diagnosis of definite neurosarcoidosis, positive nervous system histology was needed [5].

As a control group, a cohort of multiple sclerosis patients was established. Diagnosis of Multiple Sclerosis followed the recent guidelines [16, 21]. The degree of physical of disability was evaluated using the EDSS score [22]. To compare the symptom load in both cohorts, evaluation via the EDSS score that is established in multiple sclerosis was applied in both groups, i.e., in the neurosarcoidosis group as well.

Clinical parameters and examination results were derived from the written medical reports. The retrospective analysis performed in the study was approved by the local institutional ethics committee of the Friedrich-Alexander University Erlangen-Nuremberg.

### Imaging techniques

All patients underwent magnetic resonance imaging (MRI) (3 Tesla, Magnetom Trio or 1.5 Tesla Siemens Magnetom Sonata, Siemens Healthcare, Erlangen, Germany) of the brain.

#### VLSM

Two experienced investigators (K.W. and F.S.) delineated the boundaries of the hyperintense flair lesions on anonymized imaging scans using MRIcron (www.mrico.com) [23]. Both raters were blinded to clinical parameters during imaging analysis. The MRI scan and the lesion shape were transferred into stereotaxic space using the normalization algorithm of SPM8 (http://www.fil.ion.ucl.ac.uk/spm/) and the Clinical Toolbox for SPM8 (http://www.mricro.com/clinical-toolbox/spm8-scripts). Using the MR-segment-normalize algorithm of the Clinical Toolbox, the MR images were transformed to the T1 template [24]. Lesion volumes in voxels were calculated using the non-parametric mapping (NPM) algorithm. In a VLSM analysis, the lesion site was correlated with the occurrence of neurosarcoidosis using non-parametric permutation testing [25]. All lesioned voxels were included in the analysis. A false discovery rate (FDR) correction of 0.05 was applied. The peak coordinates of the involved regions are presented in Montreal Neurological Institute (MNI)-space.

### Statistical analysis

For data analysis, a commercially available statistic program (SPSS 20.0; IBM, Armonk, NY) was used. Distribution of data was tested using Shapiro–Wilk test. Data are presented as mean and standard deviation (SD) or median and interquartile range (IQR). Normally distributed patient and control data were compared using the *t* test for unpaired samples. Non-normally distributed data were compared using the Mann–Whitney *U* test. Significance was assumed for *P* < 0.05.

## Results

### Patient characteristics

The cohort included a total of 34 patients with the clinical diagnosis of definite (2), probable (19) and possible (13) neurosarcoidosis during the study period. No patient was excluded because of a comorbid other cerebral disease. A cohort of 78 randomly selected patients with multiple sclerosis served as controls.

Clinical characteristics of the participants of the study are described in Table 1. Of the 34 patients, 16 were men (47%) and 18 were women (53%). In the control group, 24 were men (31%) and 54 were women (69%). Mean age was 52 ± 9.9 years (50 ± 11.3 in the control group). Median EDSS score was 3 (IQR 1.6) and 3 (IQR 3.4) in the control group. Disease duration was significantly higher in the Multiple Sclerosis cohort (24 months, IQR 1.6 vs. 120, IQR 192).

**Table 1:** Clinical and imaging characteristics of the 34 patients with neurosarcoidosis and the 78 control patients without neurosarcoidosis, but multiple sclerosis

	Neurosarcoidosis, *n* = 34	Multiple sclerosis, *n* = 78
Age, mean ± SD; y	52 ± 9.9	50 ± 11.3
M/F	16/18	24/54
Lesion volume T2, voxels; median (IQR)	2892 ± 8126*	15,305 ± 26,750*
Definite/Probable/Possible neurosarcoidosis; *n*	2/19/13	
Multiple sclerosis type, RRMS/SPMS/PPMS; *n*		56/17/5
EDSS; median (IQR)	3 (1.6)	3 (3.4)
Disease duration, months; median (IQR)	24 (54)	120 (192)

*y* years, *IQR* interquartile range, *SD* standard deviation, *M/F* man/female ratio, *RRMS* relapsing remitting multiple sclerosis, *SPMS* secondary progressive multiple sclerosis, *PPMS* primary progressive multiple sclerosis, *EDSS* Expanded Disability Status Scale.

*Indicates significant difference using Mann–Whitney *U* test

### VLSM

Mean lesion volume was significantly higher in neurosarcoidosis than in MS controls (2892 ± 8126 voxels vs. 15,305 ± 26,750, *p* < 0.001 in the control group).

**Fig. 1:**
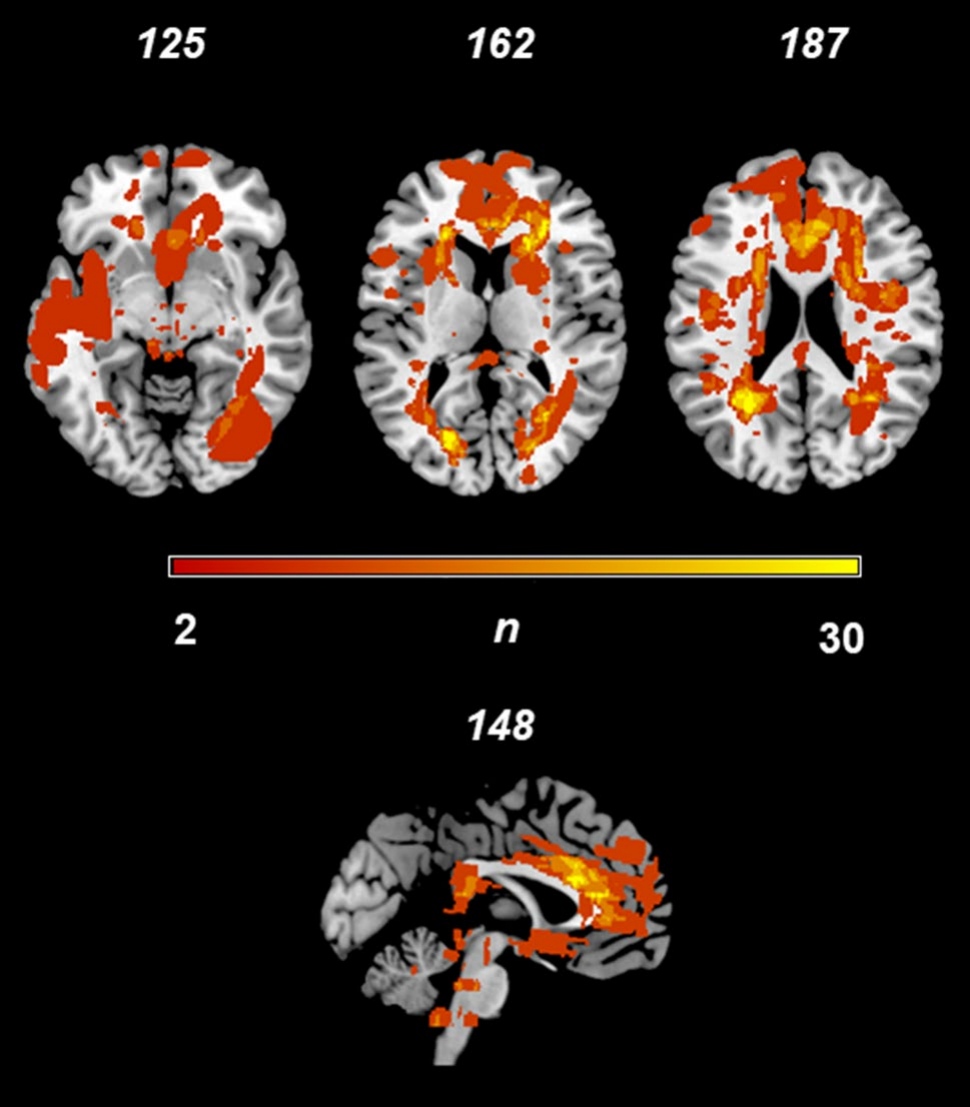
Lesion overlap map of all 34 patients with neurosarcoidosis. Lesion-overlap map in axial plane showing damaged voxels of the 34 patients with possible, probable and definite Neurosarcoidosis. Areas in red reflect damaged voxels with higher lesion overlap. *L* left hemisphere, *N* number of patients, *R* right hemisphere

**Fig. 2:**
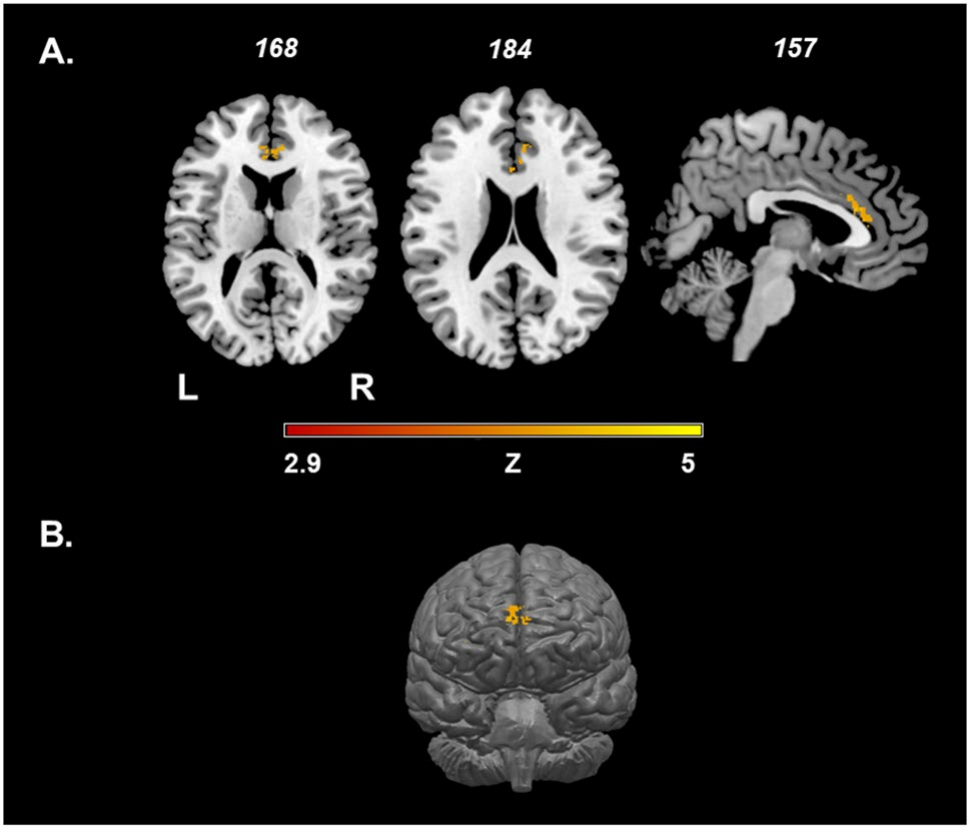
Association between occurrence of neurosarcoidosis and bilateral frontal cortical lesions. Result of the VLSM showing associations between neurosarcoidosis and damaged voxels in the bilateral frontal cortex, using the non-parametric permutation test statistics (FDR: *q* < 0.05)

Figure 1 shows the lesion overlap map of all 34 patients with neurosarcoidosis. The analysis of lesions using non-parametric permutation test shows associations between damaged voxels in the bilateral frontal cortex (Fig. 2).

## Discussion

Diagnosis of neurosarcoidosis is often very challenging. Although MRI of the brain is a cornerstone of the diagnostic workup, it mostly comprises nonspecific findings and the correlation with clinical symptoms is poor [15]. Parenchymal lesions, especially non-enhancing hyperintense white matter lesions, are the most frequent (in about 50%) abnormality found in MRI scans. However, these lesions may be indistinguishable from those of vascular or other inflammatory origin [9, 11, 13]. Systematic data on potential specific parenchymal lesion patterns in neurosarcoidosis is scarce.

As neurosarcoidosis is a serious and often devastating disease that requires therapeutic interventions that differ significantly from those for multiple sclerosis patients, it is of great importance to reliably distinguish between these two diseases and to diagnose the respective disease early and correctly [26].

Therefore, in the present study, we investigated the distribution pattern as well as other imaging characteristics in patients with neurosarcoidosis compared to multiple sclerosis using modern VLSM.

First, lesion load was significantly lower in patients with neurosarcoidosis, although the symptom load was even in our cohorts. Regarding the pathophysiology of neurosarcoidosis with a predilection for meningeal inflammation [27], we, therefore, believe that clinical symptoms are less often a result of parenchymal white matter lesions in neurosarcoidosis than in multiple sclerosis. Correlation between clinical symptoms and imaging findings is known to be poor in neurosarcoidosis [15].

Second, some striking imaging features of neurosarcoidosis, compared to multiple sclerosis, were observed. The lesion overlap (Fig. 1) demonstrated that periventricular and juxtacortical lesions are frequent in neurosarcoidosis [14, 27]. Visually, singular white matter lesions appeared to be smaller than multiple sclerosis lesions. Lesions in the corpus callosum were clearly less apparent in the neurosarcoidosis group compared to the common affection in multiple sclerosis patients, although no statistical significant difference in the Liebermeister test was observed.

Most importantly, in the Liebermeister voxelwise analysis, lesion patterns of subcortical lesions did not differ between neurosarcoidosis and multiple sclerosis patients. Therefore, although more specific imaging parameters in neurosarcoidosis would be helpful [17], it does not seem possible to draw any conclusions out of the distribution of parenchymal, subcortical lesions. Because subcortical white matter lesions are common in patients without sarcoidosis as well, it is not clear that the lesions are always related to the sarcoidosis [15].

The presence of subcortical white matter lesions is believed to be a result of two coexisting manifestations of neurosarcoidosis, and partially they seem to be of inflammatory origin [9, 11, 13, 15, 20]. In fact, enhancing mass lesions in neurosarcoidosis are frequently associated with nearby leptomeningeal involvement and are thought to represent spread of leptomeningeal disease along the perivascular spaces in many cases [9, 28]. As a hypothesis, there may be inflammatory spread along the perivascular spaces in non-enhancing white matter lesions, causing the appearance of intraparenchymal involvement, too.

Histological studies indicate that granulomatous invasion of cerebral vessel walls is not an uncommon occurrence in the disease and leads to microangiopathic lesions [9]. Microvascular changes in the central nervous system have been identified in close to 90% of cases of neurosarcoidosis at autopsy, including involvement of the small arteries, arterioles and small veins, generally without vascular complications [29]. Therefore, white matter lesions may, to some extent be attributed to vascular microangiopathic leukoencephalopathy [9, 13, 14, 27, 29].

The deep medullary vein sign, an engorgement of the deep medullary veins in brain imaging, has been proposed as a specific sign for neurosarcoidosis, However, its sensitivity is rather low (about 70%) and its role is still under debate. [17]. It is worth to note that there is the “trident sign” in cases with spinal involvement that has a significant diagnostic yield to distinguish neurosarcoidosis from other causes of myelitis [28].

Third, the lesion overlap and the voxelwise analysis demonstrated an affection of the frontal and basal cortices in neurosarcoidosis.

Meningeal inflammation, that is leptomeningeal or dural granulomatous affection, is observed in about 36% of the patients and is the second frequent imaging finding in neurosarcoidosis [15, 27]. There is a predilection for the basal areas, explaining the high rate of cranial neuropathies in neurosarcoidosis [6, 30]. The typical imaging feature is thickening and enhancement of the leptomeninges, especially around the base of the brain [15].

In leptomeningeal disease, there is a concomitant inflammatory involvement of the cortical sulci and perivascular spaces or the cisterns around the base of the brain [9, 15]. We therefore believe that the cortical, frontal lesions in our study are a result of the leptomeningeal inflammatory disease manifestation in neurosarcoidosis.

Hence, our study confirms the importance of the detection of meningeal enhancement in neurosarcoidosis. Although imaging characteristics of leptomeningeal disease are similar from that seen with tuberculosis or lymphoma involving the leptomeninges [15], clinical-radiographic features of meningeal involvement and its evolution over time in response to treatment are a helpful and quite specific tool in the diagnosis and management of neurosarcoidosis [20]. Enhancement of subcortical lesions and additional leptomeningeal enhancement patterns following Gd-DTPA administration has already been demonstrated to be useful in distinguishing sarcoidosis from multiple sclerosis [31].

Taken together, in the absence of pathologic proof, diagnosis of neurosarcoidosis remains a matter of exclusion of other diagnoses [9]. MRI aids in narrowing the differential diagnosis and can be used to demonstrate therapeutic response to immunosuppressive medication [31]. However, our study confirms previous work, that single parameters as imaging characteristics are not helpful to reliably distinguish neurosarcoidosis from MS [26].

Lab tests, CSF analysis and electrophysiological investigation further narrow the differential diagnosis [31]. The MRZ reaction (MRZR) is a polyspecific, intrathecal humoral immune response directed against the three neurotropic viruses: measles (M), rubella (R), and varicella zoster (Z), assessed using the three respective antibody.

Indices (AIs) and appears to be highly specific for MS [32]. Combined evaluation of basic CSF parameters and MRZ reaction is powerful in differentiating neurosarcoidosis from MS, with moderate to severe pleocytosis and QAlb elevation and absence of intrathecal IgG synthesis as useful rule-in parameters and positive MRZ reaction as a rule-out parameter for neurosarcoidosis [26]. However, it would be favorable in the future to develop a reliable combination of cerebrospinal fluid (CSF) parameters, specific radiological and suggestive clinical features that would obviate the need for an invasive biopsy [26].

## Limitations

Our study has limitations that should be considered in the interpretation of the results. First, we analyzed only brain imaging data. No data on clinical and imaging characteristics of spinal lesions are presented, an analysis which was beyond the scope of our study, but should be addressed in future research. Most patients were classified as probable and possible neurosarcoidosis and only two as definite neurosarcoidosis. Although a higher rate of definite neurosarcoidosis patients may have been desirable for our study, this distribution reflects real world data, as CNS biopsy is only applied with reluctance in clinical practice [33].

A strength of the present study is the voxel-wise analysis where lesion sites in the brain were correlated with the occurrence of neurosarcoidosis. Such an approach has the advantage to investigate associations between lesion sites in the brain and outcomes without having an a priori hypothesis. However, it has to be noted that the small number of patients resulted in a lower coverage by a sufficient lesion overlap to draw conclusions regarding the whole brain (see Fig. 1). In our opinion, it seems unlikely that areas which did not reach a minimum lesion overlap coverage may have yielded significant results in the statistical Liebermeister testing.

## Conclusion

In conclusion, our study demonstrated that lesion load is significantly lower in neurosarcoidosis compared to multiple sclerosis patients. Although apparently there is no propensity for lesions of the corpus callosum in neurosarcoidosis in contrast to multiple sclerosis, subcortical lesion patterns did not differ between both diseases. The VLSM analysis yielded significant associations in the bilateral frontal cortex, suggesting that leptomeningeal inflammatory disease with following cortical involvement is a quite specific feature in neurosarcoidosis.

## Data Availability

On request.

## Abbreviations

mRS: Modified Rankin scale
MRI: Magnetic resonance imaging
MS: Multiple sclerosis
VLSM: Voxel-based lesion symptom mapping

## Code availability

Not applicable/on request.
